# A framework for automated scalable designation of viral pathogen lineages from genomic data

**DOI:** 10.1038/s41564-023-01587-5

**Published:** 2024-02-05

**Authors:** Jakob McBroome, Adriano de Bernardi Schneider, Cornelius Roemer, Michael T. Wolfinger, Angie S. Hinrichs, Aine Niamh O’Toole, Christopher Ruis, Yatish Turakhia, Andrew Rambaut, Russell Corbett-Detig

**Affiliations:** 1 Department of Biomolecular Engineering, University of California Santa Cruz, Santa Cruz, CA USA; 2 Genomics Institute, University of California Santa Cruz, Santa Cruz, CA USA; 3 Biozentrum, University of Basel, Basel, Switzerland; 4 Swiss Institute of Bioinformatics, Basel, Switzerland; 5 Department of Theoretical Chemistry, University of Vienna, Vienna, Austria; 6 Research Group Bioinformatics and Computational Biology, Faculty of Computer Science, University of Vienna, Vienna, Austria; 7 RNA Forecast e.U., Vienna, Austria; 8 Bioinformatics Group, Department of Computer Science, University of Freiburg, Freiburg, Germany; 9 Institute of Ecology and Evolution, University of Edinburgh, Edinburgh, UK; 10 Molecular Immunity Unit, MRC Laboratory of Molecular Biology, Department of Medicine, University of Cambridge, Cambridge, UK; 11 Department of Veterinary Medicine, University of Cambridge, Cambridge, UK; 12 Cambridge Centre for AI in Medicine, University of Cambridge, Cambridge, UK; 13 Department of Electrical and Computer Engineering, University of California San Diego, San Diego, CA USA

**Keywords:** Phylogenetics, Taxonomy

## Abstract

Pathogen lineage nomenclature systems are a key component of effective communication and collaboration for researchers and public health workers. Since February 2021, the Pango dynamic lineage nomenclature for SARS-CoV-2 has been sustained by crowdsourced lineage proposals as new isolates were sequenced. This approach is vulnerable to time-critical delays as well as regional and personal bias. Here we developed a simple heuristic approach for dividing phylogenetic trees into lineages, including the prioritization of key mutations or genes. Our implementation is efficient on extremely large phylogenetic trees consisting of millions of sequences and produces similar results to existing manually curated lineage designations when applied to SARS-CoV-2 and other viruses including chikungunya virus, Venezuelan equine encephalitis virus complex and Zika virus. This method offers a simple, automated and consistent approach to pathogen nomenclature that can assist researchers in developing and maintaining phylogeny-based classifications in the face of ever-increasing genomic datasets.

## Abstract (web summary)

Automated and scalable designation of virus lineages based on a heuristic approach can help to determine and curate virus lineages from large genomic datasets and inform nomenclature.

## Main

Pathogen lineage nomenclature, or the designation of epidemiologically distinct groups below the level of species, is important for facilitating effective research, treatment and communication about diseases. Despite the universal importance and long history of nomenclature systems for pathogens, there remains a plurality of approaches to apply to new emerging pathogens, including using the geographic location of a variant^1,2^, specific epidemiological characteristics such as serotype^3^ or clusters of closely related viral variants^4^. The COVID-19 pandemic presented a unique challenge to these approaches. In SARS-CoV-2, a single mutation may be all that defines a new epidemiologically distinct lineage^5^. In addition, the SARS-CoV-2 genomic data are orders of magnitude greater in volume than those of extant pathogens, and are constantly growing as new data are collected^6^. The expansion of the dataset means that the SARS-COV-2 phylogeny is regularly updated^7^, necessitating further review and updates to any genotype-based lineage system.

The current solution to these challenges is the popular Pango lineage system. Pango is a genotype-based dynamic lineage nomenclature for SARS-CoV-2 (ref. ^8^). When compared with traditional nomenclature, Pango lineages often initially contain fewer samples, are less genetically distinct and are regularly updated as new genetic data are collected. These small, dynamic lineages serve a critical function in organizing genetic data for public health tracking efforts. Currently, Pango relies on manual curation and designation, including the crowdsourcing of lineage proposals on a public forum (https://github.com/cov-lineages/pango-designation). More than 2,500 SARS-CoV-2 variants have been named under the Pango system as of January 2023. The trained human eye is excellent at distinguishing new lineages of interest from groups of low-quality or contaminated isolates, but the crowdsource approach is resource intensive and vulnerable to delays and regional bias. A more objective metric to evaluate candidates for lineage designation could help to reduce this bias and streamline the lineage proposal and review process.

Here we propose a simple heuristic approach for the definition and expansion of genotype-based dynamic nomenclature systems. Our method is rooted in information theory, optimizing for the representation of sample-level haplotype information. It is efficient in application to extremely large phylogenies and produces a comprehensive hierarchy of genetically distinct lineages. Importantly, it can expand a preexisting lineage system, making the adoption of this approach for the maintenance and expansion of existing dynamic nomenclature straightforward. We, in collaboration with the Pango designation team, have implemented this system as a new input for the existing Pango lineage designation infrastructure (https://github.com/jmcbroome/autolin). In addition, as sequencing technology becomes more widely applied, both novel and extant pathogens will develop similarly dense and expanding genomic datasets. This approach will provide a scalable solution to creating and managing these dynamic lineage systems for any pathogen. In turn, these systems will allow for more effective organization and tracking of real-time pathogen evolution and outbreak events across various public health domains.

## Results

### The genotype representation index

A lineage system can be likened to a language, in which additional words, analogous to lineages, are defined for common, unique concepts to reduce the average number of words per sentence. Along these lines, an effective system summarizes a complex phylogeny into useful, distinct categories to facilitate effective analysis and communication. The lineage hierarchy is generally defined with respect to a specific rooted phylogeny, in which a number of specific ancestral nodes are designated as lineage roots (Supplementary Fig. 1). Individual samples, represented by the tips of the tree, are members of every lineage that is rooted in its inferred ancestry. To automate the construction of this hierarchy, we need some objective measure of value for putative lineage roots. One approach is to compute some importance value for every node on the tree, then iteratively construct a lineage system by selecting high-value nodes and designating them as new lineage roots. These lineages can then be presented to an end user or directly incorporated into an expanding dynamic nomenclature.

**Fig. 1:**
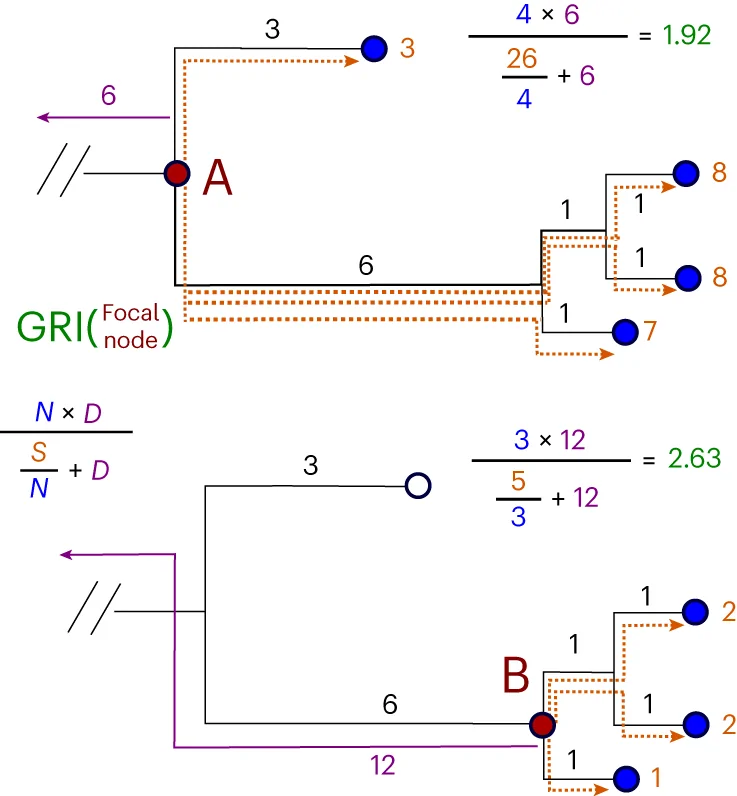
Computation of GRI. The computation of GRI values for two nodes on a small example subtree. The top panel shows the computation for node A, while the bottom panel shows the computation for node B. The base of this subtree is a total distance of 6 from the last lineage root, shown in purple. The node at the base of this subtree (A) has a total path length to descendents (*S*) of 26 and 4 total descendents (*N*), and is a total distance of 6 from the last root (*D*), leading to a GRI of 1.92. The lower child node (B) has only 3 descendents (*N*), but has a much lower path length (*S*) and a longer distance to the last root (*D*), meaning that it scores much higher at 2.63. In this case, we would choose to assign a lineage label to the lower child node (B).

To this end, we define the ‘genotype representation index’ (GRI; Fig. 1).

$$\rm{GRI}=\frac{\mathit{N}\times \mathit{D}}{\frac{\mathit{S}}{\mathit{N}}+\mathit{D}}$$

The GRI takes values with respect to a specific node on the tree, hereafter referred to as the ‘focal node’. Here *N* is the number of descendent tips from the focal node, *D* is the total branch length from the focal node to the root of the tree or previously designed parent lineage and *S* is the sum of branch lengths from the focal node to each descendent tip. In natural language, the GRI is the mean branch length position of the focal node along the ancestry paths of all its descendents, multiplied by the total number of descendents. The GRI increases both with an increasing number of descendents (*N*) and with being closely related, on average, to those descendents (lower *S*). Nodes with an overall high GRI will be closely related to many descendents, representing a group of consistently genetically distinct samples—a good choice for lineage labelling. For a mutation-annotated tree^9^, such as those used for SARS-CoV-2, the branch lengths (*D* and *S*) are in units of total mutations across the genome. However, the GRI can be computed on any rooted tree topology, as long as branch lengths are scaled by genetic distance. The GRI is high for focal nodes where descendent samples are genetically similar to one another and the focal node itself is genetically distinct from the rest of the phylogeny, desirable qualities for lineage designation^8^. The motivation behind this formulation is presented in Methods.

Autolin defines a lineage system based on the GRI by applying a simple greedy maximization algorithm. Initially, the GRI is computed for each node on the tree and the node with the highest value is chosen as a new lineage root. Additional mutually exclusive lineages are defined by disregarding all samples covered by an existing lineage label and recomputing the GRI for all remaining samples and their ancestors. To prevent the retroactive definition of lineage parents that might interfere with an existing hierarchy, we additionally disregard nodes that are directly ancestral to existing or newly added lineages. Additional hierarchical lineages are defined similarly by considering only samples within a specific existing ‘parent’ lineage. This process is repeated until a desired number of lineage labels have been defined or all available nodes fail to pass thresholds for designation. This iterative approach is not guaranteed to find the highest overall GRI lineage configuration among many possible combinations of lineages, but it scales well to millions of samples and a rapid pace of lineage updates.

### Systematic application to SARS-CoV-2 and examples

As a basic demonstration of our method, we applied the pipeline to the complete SARS-CoV-2 global public phylogenetic tree, as of 11 December 2022 from http://hgdownload.soe.ucsc.edu/goldenPath/wuhCor1/UShER_SARS-CoV-2/ (ref. ^7^). In the absence of an extant lineage system and considering all samples, the GRI-based approach assigns more than 170,000 lineages to this phylogeny. These lineages are divided into 12 levels, representing recursive levels of child lineages, with the first level being the root of the phylogeny. The majority of these lineages are small, with only 10% of designations being larger than 100 samples. Of the approximately 2,000 Pango lineages included in this phylogeny, more than 1,175 are closely matched with a GRI equivalent lineage, including the major delta and omicron lineages. Another 586 Pango lineages have a corresponding GRI-identified lineage with a Jaccard similarity of overlapping samples greater than 0.5 (Supplementary Fig. 2). The remaining unmatched 217 lineages are mostly extremely small, with more than 95% of them including <10 samples in this phylogeny, and therefore would not pass the default filters for Autolin (Supplementary Fig. 3). Overall, the systems are concordant, especially with regard to major variants. A Taxonium view of this phylogeny labelled with all levels of annotation can be explored interactively at https://taxonium.org/?protoUrl=https://media.githubusercontent.com/media/jmcbroome/lineage-manuscript/main/public-2022-12-11.independent_automated_lineages.jsonl.gz.

To evaluate the utility of our method for maintaining and expanding dynamic lineage nomenclature specifically, we applied Autolin to the same phylogeny as above, but built on the extant Pango lineages. We generated 187 new lineage designations using the default configuration parameters, which consider only samples collected in the preceding 8 weeks (Supplementary Data 1). Of these lineages, 24 were actively sampled in 11 December 2022. These active designations were highly dispersed in size, with a mean size of 82 samples and a median of 45 samples. The full report for the active designations is available in Supplementary Table 1.

**Fig. 2:**
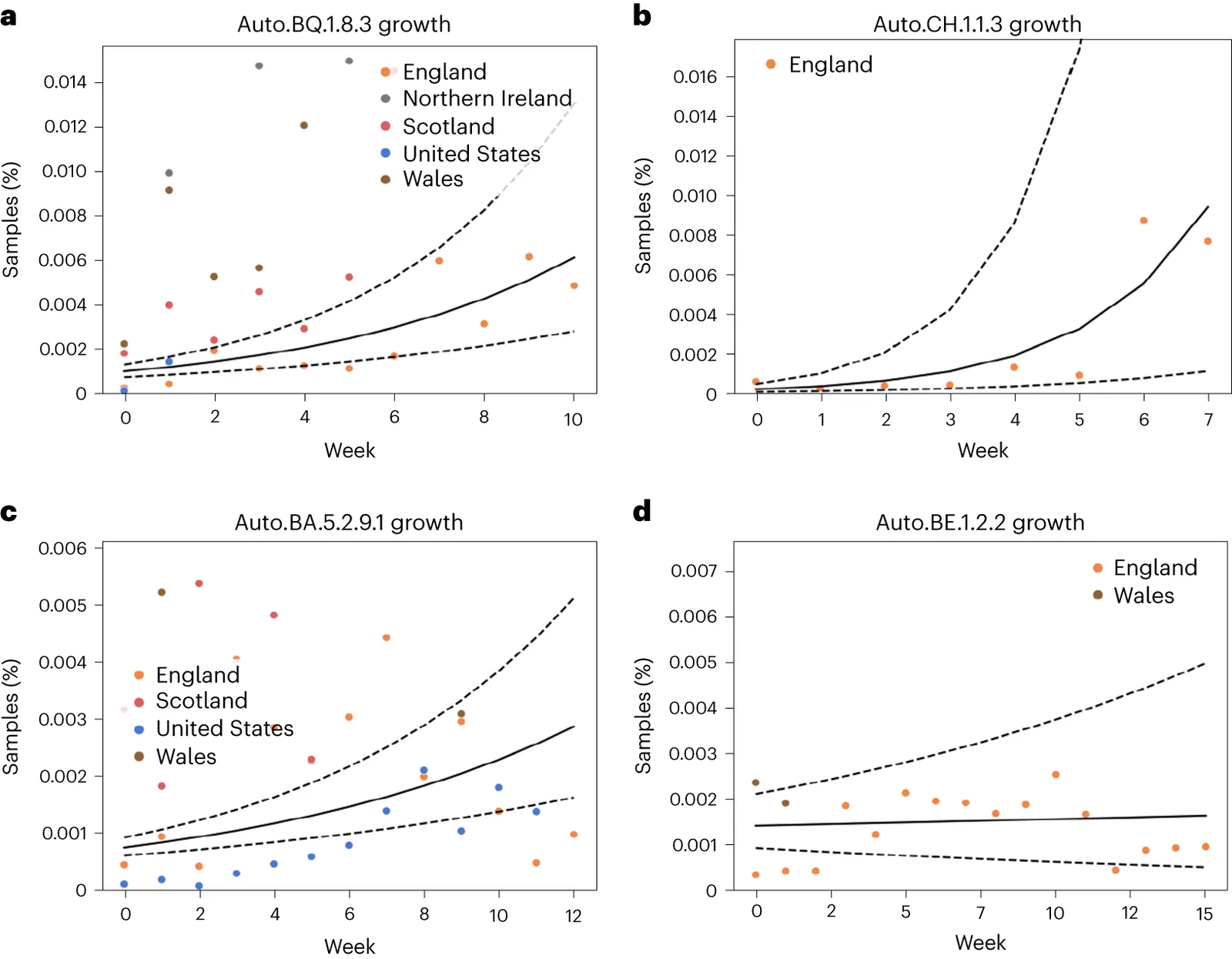
Exponential growth modelling. **a**–**d**, The four plots describe some of the lineage annotations produced by our method based on the public SARS-CoV-2 data. The solid black line is the median estimated growth trajectory, while the dashed lines represent the trajectories that would result from the lower and upper bounds of the 95% credible interval of the growth rate. The *x*-axis shows the weeks since first detection in each country. **a**, Growth trend of a lineage throughout the United Kingdom with a notably higher presence in Wales. **b**, Simple, high but low-certainty growth estimate of a lineage exclusive to England. **c**, Steady growth of an international lineage present in both the United Kingdom and the United States. **d**, A lineage which appears to have stagnated in growth and should be deprioritized for lineage labelling.

We additionally fit a simple geographically stratified exponential growth model to each active lineage (Methods, Fig. 2 and Supplementary Table 1) and obtained a 95% credible interval estimate of the rate of exponential growth. The average credible interval for the exponential growth interval was relatively large (0.07, 0.49), primarily because of the effects of limited sample sizes. Of the 24 lineages, 16 had a positive lower interval bound, which is evidence for active spread in the countries they are present in. The width of the interval is dependent on the data available; while the average estimate for our lineages is ±0.2, estimates for lineages with at least 50 total collected samples had a much narrower average value of ±0.07. All model confidence intervals are reported in Supplementary Table 1. All code for fitting and reproducing these results is available at https://github.com/jmcbroome/lineage-manuscript.

This procedure can serve to organize and prioritize lineage designations, despite suffering from high uncertainty. Figure 2 shows a small example selection of lineages and model fits in further detail. The naming schema generally matches the Pango naming schema (Supplementary Information) with the addition of an ‘auto’ prefix to indicate the origin. ‘Auto.CH.1.1.3’, while exclusive to England, exhibits a very rapid expansion in latter weeks that drives a very high, if wide, estimate of growth. Both ‘auto.BQ.1.8.3’ and ‘auto.BA.5.2.9.1’ are more international, but less consistent; the latter appears to grow consistently in the United States, but fluctuates to a much greater degree in England. Finally, ‘auto.BE.1.2.2’ is an example of a low-priority designation, with no strong evidence of positive growth. Altogether, our models can capture a diverse set of lineage trajectories and rapidly and effectively identify lineages undergoing exponential expansion.

In addition, statistics such as lineage size, associated mutations and geographic localization can be computed and reported to the user. Our update includes links to external data exploration sources such as CoV-Spectrum^10^ and Taxonium^11,12^, as well as the programmatic generation of all files requisite for the incorporation of the new designations. All code for this procedure can be found at https://github.com/jmcbroome/autolin.

### Application to other pathogens

**Fig. 3:**
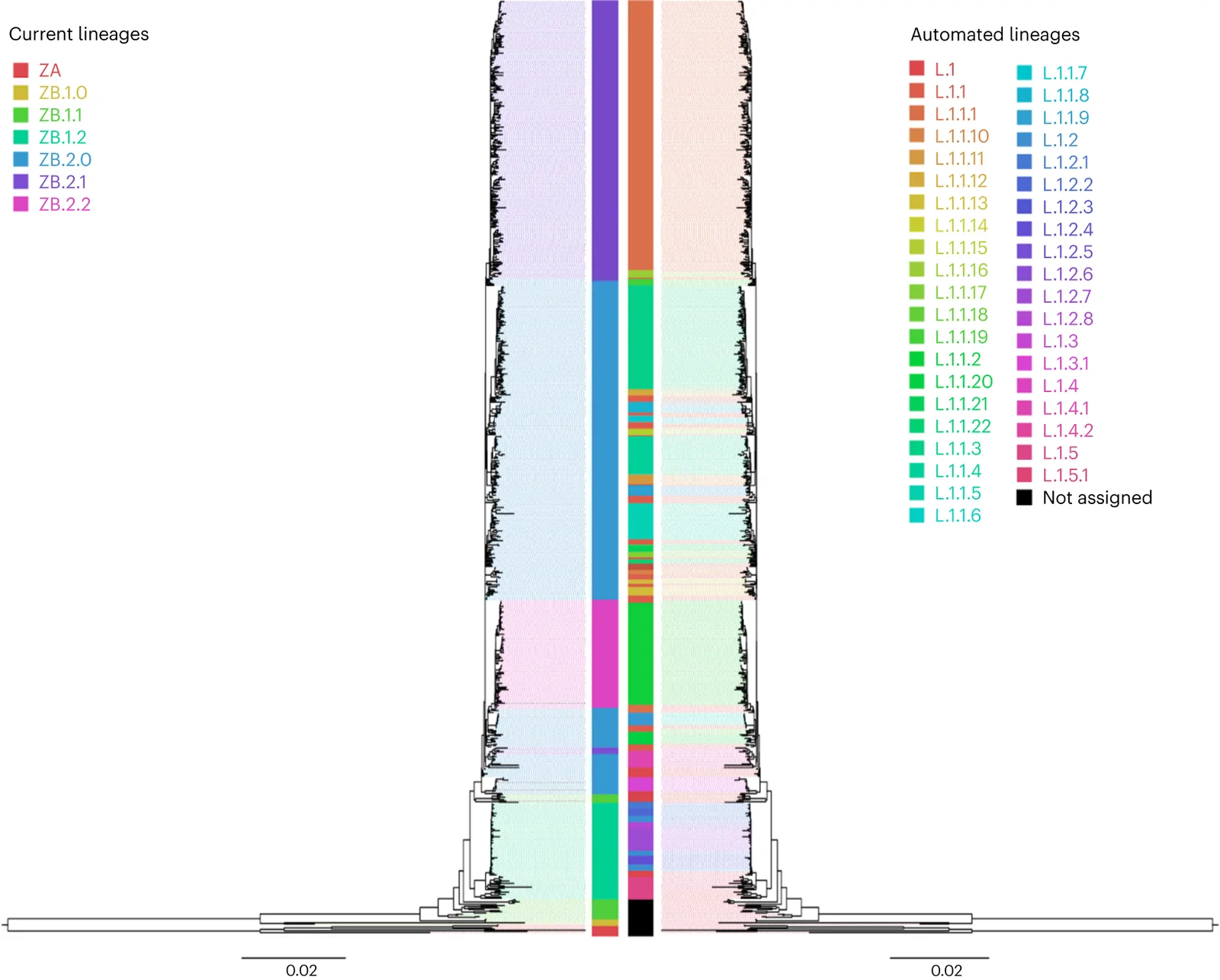
ZIKV lineages. Comparison of a published proposed lineage system for ZIKV (left tree) based on phylogenetic analyses, clustering techniques, within- and between-group pairwise genetic distances and evolutionary analyses to define genetic groups^13^ with automated lineage designation (right tree) visualized on FigTree v.1.4.4.

The GRI approach can be used to generate lineage proposals for any pathogen, with or without an existing base nomenclature. We compared our approach with a recent Zika virus (ZIKV) nomenclature proposal^13^, applying Autolin directly to their likelihood phylogeny (Methods). We find high-level concordance between the automated system and the formal nomenclature (adjusted Rand index (ARI) 0.47, *P* < 0.001; Fig. 3). The formal Zika nomenclature proposal is the result of the application of Bayesian clustering directly on aligned sample haplotypes^13^, so while this system is genotype based, it does not directly depend on the phylogeny. This may explain some of the inconsistencies between these systems, particularly as regards basal groups like ZA. However, we do see high-level concordance between these groups, particularly in the widespread ZB.2 variants.

We analysed two additional pathogens, chikungunya virus (CHIKV) and Venezuelan equine encephalitis virus complex (VEE). These phylogenies are provided as Nextstrain Auspice JSON^14^, so we used an alternative implementation of Autolin found at https://github.com/jmcbroome/automated-lineage-json designed to work with arbitrary Auspice JSON-formatted phylogenies. It is provided as both a command line interface tool and as an online Streamlit app, accessible at https://jmcbroome-automated-lineage-json-streamlit-app-3adskh.streamlit.app/. Specifically, we used the currently available nextstrain builds (CHIKV Nextstrain build 5.1 (https://nextstrain.org/groups/ViennaRNA/CHIKVnext) and VEE Nextstrain build 2.1 (https://nextstrain.org/groups/ViennaRNA/VEEnext) to generate our novel lineages.

**Fig. 4:**
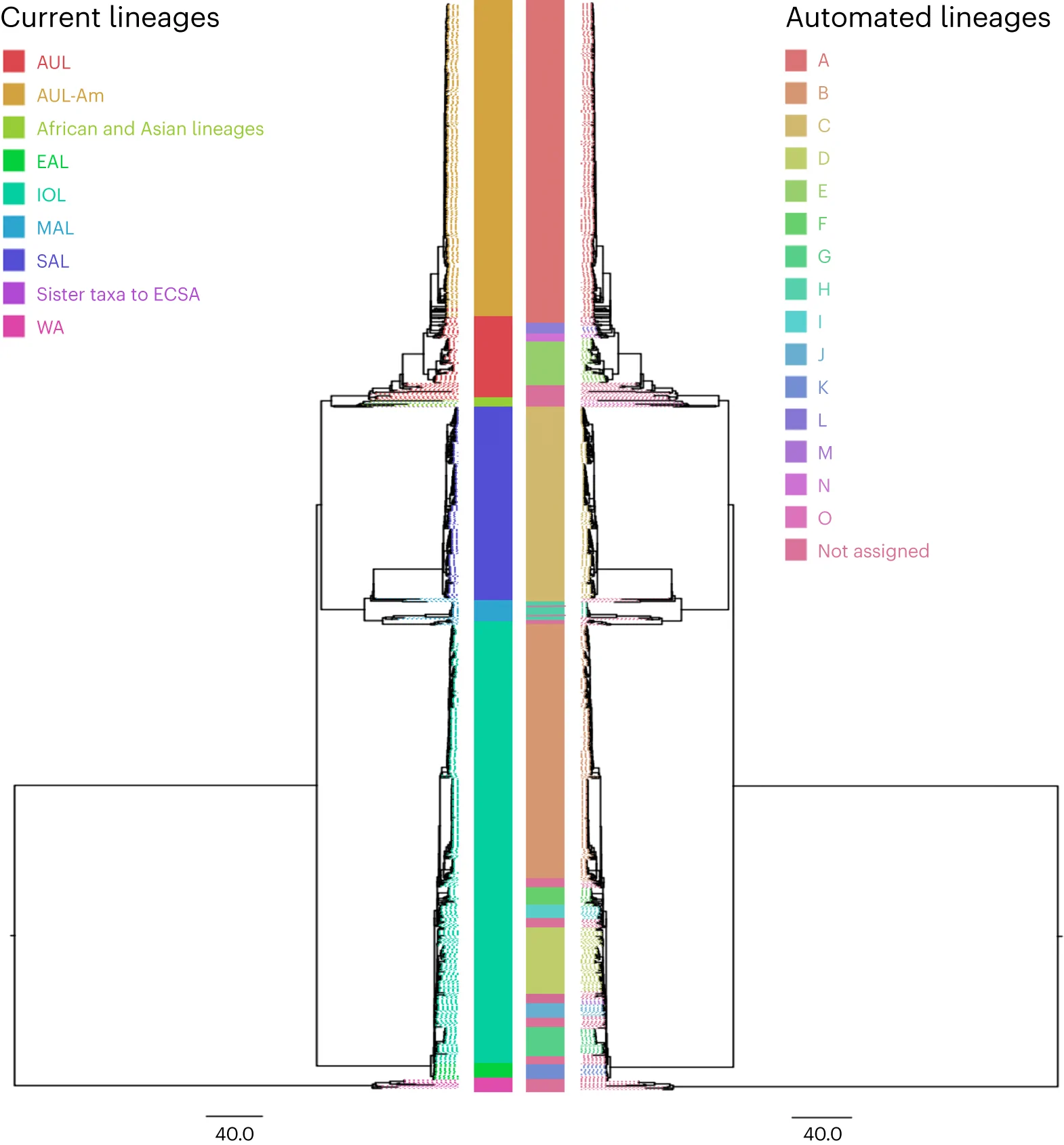
CHIKV lineages. Comparison of the geography lineage designation (left tree) with the automated lineage designation (right tree) of CHIKV, based on a tree previously generated by the Augur pipeline^23^ and visualized on FigTree v.1.4.4. The CHIKV geographic nomenclature includes AUL (Asian Urban Lineage), AUL-Am (Asian Urban Americas), EAL (East African Lineage), IOL (Indian Ocean Lineage), MAL (Middle African Lineage), SAL (South American Lineage), ECSA (East Central and South African Lineage) and WA (West African Lineage).

**Fig. 5:**
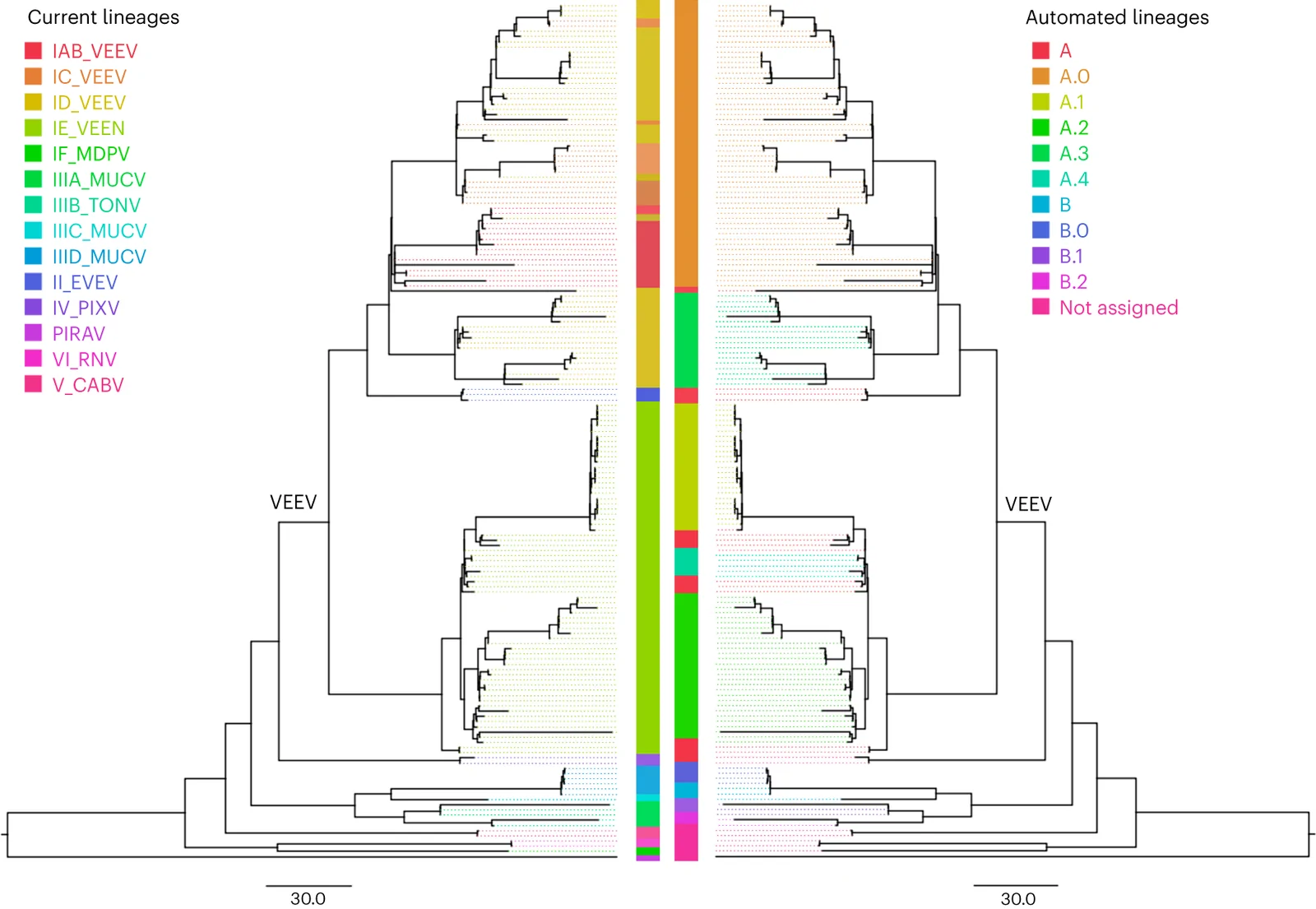
VEE lineages. Comparison of the serology subtype designation (left tree) with the automated lineage designation (right tree) of VEE, based on a tree previously generated by the Augur pipeline^23^ and visualized on FigTree v.1.4.4. According to the current nomenclature, VEE encompasses Everglades virus (EVEV), Mucambo virus (MUCV), Tonate virus (TONV), Pixuna virus (PIXV), Cabassou virus (CABV), Rio Negro virus (RNV), Mosso das Pedras virus (MDPV), Pirahy virus (PIRAV) and VEEV. The VEEV clade is labelled in the tree.

The rationale for choosing VEE and CHIKV as examples stems from their respective lineage systems. The VEE lineage system relies solely on serology, disregarding phylogenetic relationships and displaying paraphyletic groups. Conversely, the CHIKV lineage system is geographically driven and, although most often presenting monophyletic groups, relies on arbitrary thresholds to define lineages based on location. Overall, the CHIKV geographic nomenclature aligns with the automated lineage designations at its base level (ARI = 0.69, *P* = 0.018), with further breaking down of the tree in certain regions such as the Indian Ocean lineage (Fig. 4). The serology-based nomenclature of VEE, by comparison, is paraphyletic and does not represent phylogenetic lineages or clades^15,16^. We elected to present two levels of annotation, reflecting the distinction between VEE generally and the Venezuelan equine encephalitis virus (VEEV) and its subtypes. VEEV itself is successfully identified from VEE by our lineage approach at the first level of annotation (ARI = 0.9, *P* = 0.0003). However, our method was unable to reliably recapitulate VEEV serotypes at the second level of annotation (ARI = 0.28, *P* = 0.25; Fig. 5), largely because of the paraphyletic nature of the serotype-based nomenclature of VEEV.

Altogether, these examples show how this method can generate de novo lineage classification of pathogens, independent of context and consistent with human intuition. While these specific datasets may not demand a highly scalable approach to designation, they showcase the potential advantages of our methodology in mitigating user interference in lineage classification by updating biased nomenclature systems. This, in turn, enhances the possibility of epidemiological discoveries that might otherwise be overlooked. This and similar implementations of the GRI method will be able to support dynamic lineage systems for any future pathogen.

## Discussion

We have presented a new index-based method, capable of both expanding existing dynamic lineage systems and generating novel lineage designations for understudied or emerging pathogens. Originally designed for the demands of the SARS-CoV-2 pandemic, this approach can be easily applied to any rooted tree with branch lengths scaled by genetic distance.

Nonetheless, our approach does exhibit a few potential issues, shared with many lineage nomenclatures. First, it is defined with respect to a specific phylogeny. This can be problematic when attempting to maintain lineages over time, as new data are collected and the phylogeny is updated. Phylogenetic inference is naturally uncertain, and optimization of an existing phylogeny may alter lineage relationships or invalidate identified lineages. In rare cases, lineages may need to be retracted or redefined, as is the case for current Pango lineages. While these lineages are generally stable (Supplementary Information), duplicated samples, due to redundancy between data sources, can lead to inflated lineage counts or spurious lineage definitions. Appropriate filters, such as removing low-quality or duplicate samples from the input tree, will be necessary to ensure the stability and viability of these lineage systems.

In addition, rates in the variation of sequencing and contribution to public repositories of data may lead to geographical bias in the resulting designations, where poorly surveilled regions of the world are not tracked in appropriate detail. We provide methods for users to weight samples individually or as a group, including a built-in procedure to normalize the total sample weight across regions with disparate sampling. Expert review and resources will still be required to address the numerous and subtle biases that may affect the composition of data underlying these lineage systems.

Another concern is that our default approach assumes that all mutations are of equal epidemiological importance. In reality, sites associated with critical receptor binding structures or antigens are probably more important, although with substantial variation between pathogens. Our implementation of Autolin therefore includes an option to apply a weight multiplier to specific mutations or amino acid substitutions, allowing the user to leverage previous knowledge to produce more epidemiologically relevant lineage labels. For example, we have integrated information from deep mutational scan data^17^ for SARS-CoV-2 as an optional parameter in our pipeline, allowing users to upweight lineage labels that may reflect more immune-evasive variants. This and similar efforts to quantify the epidemiological importance of genetic changes will remain critical in defining and updating dynamic pathogen lineage systems.

Finally, recombination^18^ and reassortment in segmented genomes^19^ can cause some pathogen genomes to have different ancestries across their genotype, preventing their full and correct representation within a single phylogeny. These events may appear as long branches on a phylogeny^20^ or prevent the correct reconstruction of the ancestry for a specific variant. Computational methods to identify and address these events^21^ may be integrated with Autolin in the future.

SARS-CoV-2 is likely to become an endemic pathogen, similar to the influenza virus^22^. Accordingly, there is likely to be a long-term pattern of replacement of existing variants, demanding ongoing designation of new lineages for effective monitoring of pathogen diversity^8^. Investing into infrastructure to reduce manual curation will lead to long-term consistency and effectiveness of designation.

Overall, this approach for lineage designation is generic, flexible and applicable to future datasets with unclear nomenclature or expansive phylogenies. In addition, this approach can be applied outside of pathogens, allowing for fine-grained evolutionary tracking of diverse genetic datasets for medical or agricultural research and development. With global genomic sequencing on the rise, generalized toolkits for the creation and maintenance of dynamic lineage systems will be critical for future public health and research challenges.

## Methods

### Mathematical underpinnings

A lineage system can be formulated as a sender and receiver information scenario. The sender possesses the full phylogenetic tree and a lineage system *L*, while the receiver possesses only the lineage system *L* and the associated mutation paths that define each lineage. *S* may or may not be a member of any lineage within system *L*. If it is, the receiver already has all ancestry information associated with that specific lineage *L* for the sample *S*. In this scenario, we can compute how much additional information is required to specify the full ancestry of sample *S*.

A single site’s state can be represented by a finite number of bits; 2 bits to represent the state and 15 bits to represent the location, for SARS-CoV-2. Therefore, the full ancestry path of a given node *N*, which could be a sample or an internal branch, can be represented by a finite number of bits *I*, proportional to the number of mutations separating it from the root *M*.

$$I\left({N}\right)=\mathop{\sum }\limits_{1}^{M}2+15$$

Therefore, the additional information required to specify the ancestry of sample *S*, given a flat lineage system with a label at branch *B*, is

$$A\left({S,B}\right)=\left\{I\right.\left({S}\right)-I\left({B}\right){\mathrm{if}\; {S}}\in D({B})\,{\mathrm{otherwise}}$$

where *D*(*B*) is the set of samples descended from a labelled branch *B*.

We further refined this concept to represent instead the average proportion of information about sample *S* conveyed by a lineage *B*. This normalization procedure ensures that all samples are treated equally and that the lineage itself is an effective representation of the member samples. By normalizing to total distance, a cluster of samples near the reference will be treated the same as a similar group of samples positioned further from the root, given that the groups are similarly distinct from their last respective lineage labels. We therefore compute the following:

$$P\left(S,B\right)=\left\{\frac{I\left(S\right)-(B)}{I(S)}{\mathrm{if}\; S}\in D\left(B\right),\;1\;{\mathrm{otherwise}}\right.$$

We extend this to compute the total amount of information for a system of multiple lineage branches *B*. These may be hierarchically arranged, where a single sample is descended from multiple, nested lineage labels *B*; in this case, the minimum value is taken.

$$Y=\left\{{B}_{1},\ldots ,{B}_{n}\right\}$$

$$O\left(Y\right)=\sum _{S\in T}\min (\left\{P\left(S,B\right):B\in Y\right\})$$

When adding a new branch *B* to this system, we can compute the difference in overall information represented by this addition. Adding a lineage *B* will always either reduce *O*(*Y*) or leave it the same, as any altered values summed to *O*(*Y*) are replaced by a smaller value.

$$Y^{\prime} =\left\{{B}_{1},\ldots ,{B}_{n+1}\right\}$$

$$O({Y}^{{\prime} })\le O(Y\;)$$

The difference between *O*(*Y*′) and *O*(*Y*) can be computed as the sum of differences in *P*(*S*,*B*) for all samples where *B*_*n*+1_ is the terminal lineage of that sample, that is, where *P*(*S*,*B*_*n*+1_) is the minimum value of *P*(*S*,*B*) for all *B*. For all other values, *P*(*S*,*B*) is identical and therefore can be disregarded.

$$O\left(Y\right)-O\left({Y}^{{\prime} }\right)=\sum _{S\in T}(\left(\left\{P\left(S,B\right):B\in Y\right\}\right)-P(S,{B}_{n+1})$$

Our goal is to choose the value of *B*_*n*+1_ that maximizes the overall difference. The first term is constant with respect to *B*_*n*+1_, so the difference between choices of *B*_*n*+1_ is defined by the remaining term.

$$C=\sum _{S\in T}\min (\left\{P\left(S,B\right):B\in Y\right\})$$

$$O\left(Y\right)-O\left({Y}^{{\prime} }\right)=C-\sum _{S\in T}P(S,{B}_{n+1})$$

Samples *S* where *B*_*n*+1_ is not the terminal (most recent and closest on the ancestral line) lineage will be valued the same regardless of the choice of terminal lineage. By splitting the sets accordingly, we can further divide the term into constant and variable components with respect to *B*_*n*+1_. All samples on phylogeny *T* are either a terminal descendent *D*_t_ of *B*_*n*+1_ or not, forming mutually exclusive sets that combine to equal all samples on phylogeny *T*. This allows us to divide the sum into two components.

$$\left(S\notin {D}_{\mathrm{t}}\left({B}_{n+1}\right)\right)=S\in T$$

$$\left(S\notin {D}_{\mathrm{t}}\left({B}_{n+1}\right)\right)\bigcap \left(S\in {D}_{\mathrm{t}}\left({B}_{n+1}\right)\right)={{\varnothing }}$$

$$\sum _{S\in T}P\left(S,{B}_{n+1}\right)=\sum _{S\notin {D}_{\mathrm{t}}({B}_{n+1})}P\left(S,{B}_{n+1}\right)+\sum _{S\in {D}_{\mathrm{t}}({B}_{n+1})}P\left(S,{B}_{n+1}\right)$$

*P*(*S*,*B*_*n*+1_) is valued as 1 when *S* is not descended from branch *B*; therefore, the sum of *P*(*S*,*B*_*n*+1_) over the set of *S* not descended from *B*_*n*+1_ is equal to its size, and we can substitute this value in our equation.

$$S\notin {D}_{\mathrm{t}}\left({B}_{n+1}\right)$$

$$\sum _{S\notin {D}_{\mathrm{t}}({B}_{n+1})}1={\rm{\Vert }}S\notin {D}_{\mathrm{t}}({B}_{n+1}){\rm{\Vert }}$$

$$\sum _{S\in T}P(S,{B}_{n+1})=\Vert S\notin {D}_{\mathrm{t}}({B}_{n+1})\Vert +\sum _{S\in {D}_{\mathrm{t}}({B}_{n+1})}P(S,{B}_{n+1})$$

$$O\left(Y\right)-O\left({Y}^{{\prime} }\right)=C-{\rm{\Vert }}S\notin {D}_{\mathrm{t}}\left({B}_{n+1}\right){\rm{\Vert }}-\sum _{S\in {D}_{\mathrm{t}}({B}_{n+1})}P\left(S,{B}_{n+1}\right)$$

where *D*_t_ is the set of samples for which *B* is the terminal lineage. As all samples in *D*_t_(*B*) are necessarily members of *D*(*B*), this is equivalent to the following:

$$O\left(Y\right)-O\left({Y}^{{\prime} }\right)=C-{\rm{\Vert }}S\notin {D}_{\mathrm{t}}\left({B}_{n+1}\right){\rm{\Vert }}-\sum _{S\in {D}_{\mathrm{t}}({B}_{n+1})}\frac{I\left(S\right)-I({B}_{n+1})}{I(S)}$$

We can simplify the magnitude term by exchanging it for terms that are constant and dependent on the set *D*_t_(*B*_*n*+1_) and combining the relevant components with the existing constant and sum.

$${\rm{\Vert }}S\in T{\rm{\Vert }}-{\rm{\Vert }}S\in {D}_{\mathrm{t}}\left({B}_{n+1}\right){\rm{\Vert }}={\rm{\Vert }}S\notin {D}_{\mathrm{t}}({B}_{n+1}){\rm{\Vert }}$$

$$O\left(Y\right)-O\left({Y}^{{\prime} }\right)=C-{\rm{\Vert }}S\in T{\rm{\Vert }}+{\rm{\Vert }}S\in {D}_{\mathrm{t}}\left({B}_{n+1}\right){\rm{\Vert }}-\sum _{S\in {D}_{\mathrm{t}}\left({B}_{n+1}\right)}\frac{I\left(S\right)-I({B}_{n+1})}{I(S)}$$

$${C}^{{\prime} }=C-{\rm{\Vert }}S\in T{\rm{\Vert }}$$

$$O\left(Y\right)-O\left({Y}^{{\prime} }\right)=C^{\prime} +{\rm{\Vert }}S\in {D}_{\mathrm{t}}\left({B}_{n+1}\right){\rm{\Vert }}-\sum _{S\in {D}_{\mathrm{t}}\left({B}_{n+1}\right)}\frac{I\left(S\right)-I({B}_{n+1})}{I(S)}$$

Both the magnitude term and the sum are dependent on the number of samples descended from *B*_*n*+1_, so we can replace the magnitude term by subtracting one from the second term on each step through the sum.

$$\sum _{S\notin {D}_{\mathrm{t}}\left({B}_{n+1}\right)}1={\rm{\Vert }}S\notin {D}_{\mathrm{t}}({B}_{n+1}){\rm{\Vert }}$$

$$\sum _{S\in {D}_{\mathrm{t}}\left({B}_{n+1}\right)}1-\sum _{S\in {D}_{\mathrm{t}}\left({B}_{n+1}\right)}\frac{I\left(S\right)-I({B}_{n+1})}{I(S)}=\sum _{S\in {D}_{\mathrm{t}}\left({B}_{n+1}\right)}1-\frac{I\left(S\right)-I({B}_{n+1})}{I(S)}$$

$$O\left(Y\right)-O\left({Y}^{{\prime} }\right)=C^{\prime} +\sum _{S\in {D}_{\mathrm{t}}\left({B}_{n+1}\right)}1-\frac{I\left(S\right)-I({B}_{n+1})}{I(S)}$$

We can then further simplify the second term.

$$O\left(Y\right)-O\left({Y}^{{\prime} }\right)=C^{\prime} +\sum _{S\in {D}_{\mathrm{t}}\left({B}_{n+1}\right)}\left(1-\frac{I({B}_{n+1})}{I(S)}\right)$$

$$O\left(Y\right)-O\left({Y}^{{\prime} }\right)=C^{\prime} +\sum _{S\in {D}_{\mathrm{t}}\left({B}_{n+1}\right)}\frac{I({B}_{n+1})}{I(S)}$$

In practice, we often track the information about the branch *I*(*B*) and the distances to the descendent samples *S* from that branch *B* as explicit quantities.

$$F\left(S,B\right)=I\left(S\right)-I(B)$$

$$O\left(Y\right)-O\left({Y}^{{\prime} }\right)=C^{\prime} +\sum _{S\in {D}_{\mathrm{t}}\left({B}_{n+1}\right)}\frac{I({B}_{n+1})}{F\left(S,{B}_{n+1}\right)+I({B}_{n}+1)}$$

This equation is the basis of the Autolin heuristic, which is a computationally practical representation of lineage information content.

### GRI and the Autolin algorithm

We want to avoid computing the set of samples *D*_t_ for each node *B* on the tree explicitly, as this requires either repetitive traversal or storing large arrays of values. We also choose to consider only branches *B*_*n*+1_ where *D*_t_(*B*_*n*+1_) equals *D*(*B*_*n*+1_)—that is, branches with no existing lineages specific to some of its descendents—to prevent the retroactive definition of parent lineages and ensure the algorithm proceeds with straightforward, hierarchical levels of annotation at each step. The only dependent term on this set of samples *S* is *F*(*S*,*B*_*n*+1_). We therefore replace this term by dynamically computing the mean *F*(*S*,*B*_*n*+1_) for all samples *S* and multiplying the entire equation by the number of descendents, meaning we have to compute this overall equation only once. While this is not exactly equivalent to the sum, except under special conditions, it is strongly correlated with it and can reduce the effect of outlier samples on the overall computation. We also disregard the constant term when comparing values of *D*(*B*_*n*+1_) for this algorithm.

$$O\left(Y\right)-O\left({Y}^{{\prime} }\right)\propto \left|D({B}_{n+1})\right|\cdot \left(\frac{I({B}_{n+1})}{\left(\frac{{\sum }_{S\in D({B}_{n+1})}F(S,{B}_{n+1})}{\left|D({B}_{n+1})\right|}\right)+I({B}_{n+1})}\right)$$

This allows us to track only three values for each node—the sum of distances *F*(*S*,*B*), the number of descendents |*D*(*B*)| and the information of the branch *I*(*B*), and perform only a single computation. The sum of *F*(*S*,*B*) and the number of descendents |*D*(*B*)| can both be dynamically computed by a single reverse postorder traversal of the tree and stored as single float values.

$$\begin{array}{l}{\mathrm{Sum}}\left(B\right)=\left\{{\mathrm{branch}}\; {\mathrm{length}}\; {\mathrm{of}\; B\; \mathrm{if}\; \mathrm{children}}\left(B\right)\right.\\\left.\qquad\quad={{\varnothing }}\sum _{C\in {\mathrm{children}}(B)}{\mathrm{sum}}\left(C\right){\mathrm{otherwise}}\right.\end{array}$$

$$\mathrm{Count}\left(B\right)=\left\{1\;{\mathrm{if}}\; {\mathrm{children}}\left(B\right)={{\varnothing }}\sum _{C\in {\mathrm{children}}(B)}{\mathrm{count}}\left(C\right)\mathrm{otherwise}\right.$$

*I*(*B*) can be dynamically computed by a single forward traversal, as the branch length *I*(*B*) is equal to the branch length of *B* plus the information of its parent. We perform one pass to compute the sum and count values, and we track *I*(*B*) on the forward pass where candidate nodes are evaluated. With these values for each node, we can compute the following:

$$\mathrm{GRI}\left(B\right)=\frac{\mathrm{count}(B)\cdot I(B)}{\frac{\mathrm{sum}(B)}{\mathrm{count}\left(B\right)}+I(B)}$$

Notationally, we use single letters to refer to the values of these functions for a branch *B*.

$$S=\mathrm{sum}(B)$$

$$N=\mathrm{count}\left(B\right)$$

D=I(B)

$$\mathrm{GRI}=\frac{N\cdot D}{\frac{S}{N}+D}$$

This final equation is the GRI heuristic we use to select our lineages. It does not require identifying the explicit set of descendent samples *D*(*B*), which for large phylogenies either requires storing large vectors in memory or repeated tree traversal, instead using single values for the sum and count. It also has useful properties; it can never have a higher value than *N*, limiting the effect of extremely long branches, and approaches 0 as *S* becomes large, where the lineage proposal would be a poor representative of its descendents.

$$\frac{N\cdot D}{\frac{S}{N}+D}=N$$

$$\frac{N\cdot D}{\frac{S}{N}+D}=0$$

In the simplest case, the construction of a lineage system will involve the stepwise addition of lineage labels. Finding the overall system that maximizes the relative gain for multiple simultaneous lineage definitions is excessively complex and unscalable for systems of more than a handful of lineages, owing to the extremely high number of possible combinations of lineage labels to evaluate. However, a system of arbitrary size can be constructed efficiently through a simple greedy stepwise algorithm, where the best choice for each step is taken without regard for the impact on potential future choices. Therefore, our implementation computes this metric for every node on the tree, assigns a new lineage at the highest value node and then repeats this process until no candidates pass minimum thresholds set by the user. ‘Serial’ or non-overlapping lineages, where

$$D\left({L}_{1}\right)\cap D\left({L}_{2}\right)={\rm{\varnothing }}$$

can be assigned by repeating the minimization procedure while disregarding all samples that are a member of existing lineages. This can be repeated until some minimum percentage of samples are contained within some set *D*(*L*).

‘Hierarchical’ or nested lineages, where

$$D({L}_{2})\subseteq D({L}_{1})$$

can be assigned by treating *L*_1_ as the root of the tree, with ancestry information conveyed with respect to it. There are no other types of lineage relationship, as a rooted phylogenetic tree is a directed acyclic graph and lineages are always defined as a monophyletic clade. It is not possible for two clades to partially overlap when they are defined by internal nodes on a fixed phylogenetic tree.

### Restricting generated lineages

There is one obvious failure case with this model; if the number of lineage labels *B* is not limited or penalized, every node in the tree can be given individual labels, reproducing the original phylogeny and all accompanying information exactly in the lineage system. However, this degenerate case is not desirable, as the goal of lineage systems is generally to compress phylogenetic information to a more manageable set of groups while keeping key elements. Two simple restrictions are a minimum lineage size and a minimum distinction from the parental lineage or root.

To require a minimum number of samples to be represented by a putative lineage label, we define a minimum *m* and we subtract the weighted mean information represented by a theoretical set of *m* samples with the same path length distribution from the true information distribution for the node. If the net information represented is negative, then we reject this node as a candidate for a new lineage definition. We define the following inequality:

$$\frac{(N-m)\cdot D}{\frac{S}{N}+D} > 0$$

Essentially, we require that *N* > *m*, where *m* is a user-selected parameter, to define a new lineage. Setting this to some positive value will produce only proposed lineages that convey some information about at least that many leaves.

Similarly, we can set a minimum distinguishing distance from the subtree root or parent lineage. Often lineage designation systems require some number of distinguishing mutations for a new sublineage. We therefore define the following inequality:

$$\frac{N\cdot (D-p)}{\frac{S}{N}+D} > 0$$

When *p* < *D*, this value is negative and we reject this candidate node. Setting this to 2, for example, will produce only lineages that convey at least two unique mutations distinct from the parent lineage or tree root. Combining both of these filters, we reject nodes where either or both of these inequalities are not passed. Together, this allows automatic proposals to fulfil standard conditions required by lineage nomenclature review groups.

### Additional parameters

Our pipeline implementation includes a substantial set of configurable parameters. These include minimum lineage size and minimum distinction, as outlined above. We also can simply threshold on the GRI itself, ignoring marginal designations that contain relatively little additional information.

Notably, we can additionally incorporate arbitrary sample-level weighting. This allows our lineage system to prioritize effective representation of high-interest samples. *R*(*S*), below, is a function representing the ‘importance’ of sample *S*. This might be high for a sample *S* from an under-sequenced region, or lower for a sample *S* from a heavily sequenced time or place.

$$W=\sum _{S\in D(B)}R(S)$$

$$\frac{W\cdot D}{\frac{S}{N}+D}$$

Samples from regions that contribute a small percentage of all samples will have substantially higher weights than ones from regions that contribute a large percentage of sequences, although all samples will have a weight greater than 1 under this schema. This is just one potential weighting schema for handling geographic sequencing bias, and the user can define any schema and set weights on a per-sample basis.

Similar concepts can apply to computing path lengths—we may consider only part of the haplotype, or assign additional weight to specific mutations of interest that we want our lineage system to prioritize representing. We provide options for the user to select genes of interest for representation, as well as the ability to ignore mutations that do not change the amino acid content of proteins and represent coding haplotypes only.

We also provide arbitrary weighting schema for mutations of interest, similar to samples. As an example, we provide a parameter that heavily weights mutations that are predicted to increase vaccine escape^17^. This parameter multiplies the escape weight value estimated by the Bloom lab calculator by the user’s parameter and adding 1. In this schema, mutations that are not predicted to contribute to immune escape have a weight of 1, while mutations that do contribute have a weight greater than 1 that is proportional to the strength of escape conferred. The resulting lineage system is more likely to include designations that have a change in immune escape. This is just one possible schema, and the user can define weights on a per-mutation basis in our implementation.

All parameters and configuration information used in the production of these results can be found in Supplementary Data 1.

### Sorting and prioritizing novel lineages with simple Bayesian growth modelling

In some cases, curators may prefer to designate a smaller number of lineages that are of higher apparent epidemiological impact, to improve the average impact and simplicity of the lineage system. In this case, our approach can be applied to identify many individual lineage candidates, which can then be filtered and prioritized according to lineage-level statistics. While many simple filters we support, such as the number of countries a lineage has been detected in, are simply applied to the tabular report, we do also provide a more informed sorting schema based on lineage growth.

To sort putative lineages for manual inspection after the initial designation procedure, we fit a geographically stratified exponential growth model to each proposed lineage using Markov Chain Monte Carlo. Bayesian methods of this type are appropriate for inference with small, noisy datasets, as the uncertainty in the model is directly quantified. Our simplified Bayesian growth model is a geographically stratified estimate of a fundamental rate of exponential growth over a weekly time series. We model the true percentage *P* of cases in country *C* that are of lineage *L* as increasing in an approximately exponential fashion. This is appropriate for newly emerging lineages that consist of a small percentage of total cases in any country where they are found but are successfully spreading. Each data point consists of the total number of samples from lineage *L* found in a specific country during a specific week. We assume that the inherent exponential growth coefficient for *L* is shared across all countries in which it is found and combine all data points across countries and times for each lineage. The first week that any sample from lineage *L* was found in country *C* is treated as the initial timepoint (*t* = 0) for data from that country.

We do not directly observe the true percentage of cases *P* that are of lineage *L*. Instead, some number *N* of all cases are sequenced, and we observe some number *X* of these samples to be lineage *L*. As the number of cases is much larger than the number of samples, we can model this process as a binomial sampling procedure with *N* trials and a probability of success being the true percentage *P*.

Our Bayesian model combines both this sampling procedure and the exponential growth model to yield a posterior distribution of growth values that can explain the behaviour of lineage *L*. Often these distributions are wide, owing to sparse sampling and noise over few data points. In addition, some lineages may not fit an exponential growth model at all, owing to being outcompeted by newly introduced lineages or simple epidemiological noise, leading to highly variable estimates of growth. Accordingly, we compute the 0.025 and 0.975 quantiles (95% CI) for this distribution for each lineage *L* and sort the output by the lower quantile. Lineages with a large positive value for the lower quantile will reliably resemble a high exponential growth model and are more likely to be of epidemiological concern.

This model is extremely simple compared with standard epidemiological models owing to the constraints of available data and necessary speed. A more complex model would require metadata unavailable for most genome sequences, such as patient symptoms and other protected health information. Instead, this model only considers the change in the proportion of sequences from different areas belonging to a given lineage over time. Results from this approach will accordingly suffer from variance from differences in national health policy and sequencing strategy with respect to patient symptoms. It additionally may be biased by the presence and distribution of competing variants across different localities, as well as local vaccination levels and overall population susceptibility. Because of these limitations, this model does not directly inform the initial designation of lineages, but instead serves as an optional out-of-the-box solution for users to highlight putative lineages that may be of immediate and critical public health importance without substantially adding to the overall compute time for the pipeline.

All code for our modelling and reporting process can be found at https://github.com/jmcbroome/lineage-manuscript and https://github.com/jmcbroome/autolin.

### Applying Autolin to other pathogens

To validate that this method can be applied to pathogens other than SARS-CoV-2, we selected two Nextstrain instances for CHIKV and VEE, which are currently classified based on their geography and serology, respectively. We applied our generalized implementation (https://github.com/jmcbroome/automated-lineage-json) under default settings for the Auspice JSON files of each virus (CHIKV Nextstrain build 5.1 available at https://doi.org/10.5281/zenodo.7514289 and VEE Nextstrain build 2.1 available at https://nextstrain.org/groups/ViennaRNA/VEEnext (https://doi.org/10.5281/zenodo.7524848)) to obtain lineage assignments. These Nextstrain JSON files were generated by the Augur^23^ pipeline (Nextstrain-Augur v19.1.0, Treetime v0.9.4, IQ-TREE v2.2.0). We then downloaded the Nexus file with annotations from the new JSON file from Nextstrain and visualized and compared the annotations using FigTree v.1.4.4. Tree figure comparisons were made by extracting them in pdf format as shown in FigTree, mirrored and aligned on a photo-editing software. Taxon labels were coloured according to the lineage assignment and were replaced with bars representing the colour of the lineage for best visualization.

There was no available Auspice build for the ZIKV nomenclature^13^. We therefore had to construct a mutation-annotated tree (MAT) to make a file compatible with Autolin. We obtained the phylogeny directly from the authors and sample names and lineage assignments from their Supplementary Table 3. We downloaded sample sequences using the Entrez API and aligned them to the same Zika reference (KJ776791) used in a previous study^13^ with Minimap2^24^ to produce a variant call format (VCF) file. We then combined this VCF file and their likelihood phylogeny into a MAT with likelihood branch lengths using UShER^9^. We applied Autolin to this MAT with a minimum lineage size of 3 and a minimum distinction (distance in total branch length from the last annotated lineage) of 0. Finally, we extracted the new lineage annotations for each sample using matUtils^7^. Strictly, the mutations inferred did not affect this process, as the GRI is dependent on the branch lengths, but constructing the MAT was necessary to make the data compatible with the Autolin implementation of the GRI. All code to reproduce this process can be found at https://github.com/jmcbroome/lineage-manuscript. Figure 5 was produced as described above with FigTree.

We compared the automated lineage assignments with the previous nomenclature using the ARI. We used the ARI instead of identifying best-match lineages via the Jaccard as we did for the comparison directly to Pango because of the relative flatness of the relevant systems; ARI is well suited to comparing two discrete sets of labels, disregarding hierarchy. While Autolin may be much finer grained at the terminal level compared with a given nomenclature, we consider it to be a success if the existing nomenclature is largely captured by some higher level of Autolin labels, indicating that Autolin has identified these relevant groups along with potentially relevant subgroups. Therefore, we compared the set of terminal lineages for the preexisting systems for each pathogen individually with each level of annotation produced by Autolin, disregarding metaclusters of related annotations on that level, and noted the highest value. A similar process for Pango would require dividing Pango into several hierarchical levels, along with the automated system, and performing a large number of pairwise comparisons, which in turn reduces our power to detect statistically significant commonalities. For these other pathogens, with shallower lineage systems, this process is more tractable. To establish a null distribution, we randomly selected nodes in the amount of the number of categories found for each annotation to create a distribution of random ARIs to evaluate the robustness of the method. By selecting random nodes within the tree and taking their descendents to construct our null comparisons, we account for natural correlation from the tree structure, while the ARI itself accounts for variations in group sizes. We then compute the percentile of the true ARI of our lineage proposals against the existing nomenclature from the permuted null distribution, yielding the reported *P* values. All code for this can be found at https://github.com/jmcbroome/lineage-manuscript.

### Reporting summary

Further information on research design is available in the Nature Portfolio Reporting Summary linked to this article.

## Supplementary information

## Data Availability

All SARS-CoV-2 phylogenies are available from http://hgdownload.soe.ucsc.edu/goldenPath/wuhCor1/UShER_SARS-CoV-2/. Processed and raw CHIKV, VEE and Zika phylogenies are available at https://github.com/jmcbroome/lineage-manuscript/. Interactive Nextstrain views of the phylogeny for CHIKV can be found at https://nextstrain.org/groups/ViennaRNA/CHIKVnext and for VEE at https://nextstrain.org/groups/ViennaRNA/VEEnext. Interactive views for the SARS-CoV-2 and Zika phylogeny may be obtained by downloading the protocol buffer (pb) files from https://github.com/jmcbroome/lineage-manuscript/ and uploading them to https://taxonium.org/. The list of Zika accessions referenced in this paper are available in the supplement of ref. ^13^ (https://academic.oup.com/ve/article/8/1/veac029/6555351#351081937) with additional information at https://github.com/seabrasg/zika_diversity.

## Code availability

All software for lineage designation and analysis is available on GitHub, at https://github.com/jmcbroome/autolin (ref. ^25^), https://github.com/jmcbroome/lineage-manuscript (ref. ^26^) and https://github.com/jmcbroome/automated-lineage-json (ref. ^27^).
