# Supplementary material for: A framework for automated scalable designation of viral pathogen lineages from genomic data
Source: Nat Microbiol. 2024 Feb 5;9(2):550–60. doi: 10.1038/s41564-023-01587-5 (PMC10847047; doi:10.1038/s41564-023-01587-5)
Supplement: Supplementary file 1 — Supplementary Discussion 1 and 2, and Supplementary Figs. 1–3. [file 41564_2023_1587_MOESM1_ESM.pdf]

nature microbiology

Analysis

https://doi.org/10.1038/s41564-023-01587-5

A framework for automated scalable designation of viral pathogen lineages from genomic data

In the format provided by the authors and unedited

Supplementary Material

S1: Lineage Naming Syntax

Autolin uses a simplified version of the Pango lineage schema (https://www.pango.network/the-pango-nomenclature-system/statement-of-nomenclature-rules/). Autolin disposes with aliases, which exist primarily to compress names for the convenience of human readers, but otherwise follows the schema. To summarize, after initial lineages are designated with letters in alphabetical order (A, B, etc), sublineages are designated by a period and a number indicating the order of sublineage designation appended to the parent designation. For example, the first sublineage of A is A.1, while the third sublineage of B is B.3. Further sublineages are follow in the same fashion, with the fourth sublineage of A.1 being A.1.4, and so on. In this way, each label represents its own lineage ancestry- a sample in A.1.4 is a member of A.1 as well as A. Autolin also prepends “auto.” to any proposed new lineage names by default, clarifying to the user that these lineages are newly added by Autolin. Users may replace these interim names with more readable, pathogen-appropriate alternatives after using Autolin. Notably, we optionally provide Pango-style lineage compression as a part of our designation pipeline output.

S2: Lineage Stability Analysis

To identify any potential issues with respect to the stability of lineages defined by Autolin with respect to the SARS-CoV-2 tree, we identified new lineages via Autolin and tracked the phylogenetic placements of the associated samples over the following month. We began with the global public phylogeny as of 2023-04-01, and computed a set of 65 new lineage annotations with Autolin. The new designations covered 8,951 samples, with a median size of 50 samples per lineage and the majority being sub-variants of the XBB lineage. We then transferred these designations to each successive daily tree through 2023-04-30 using matUtils annotate and tracked lineage membership of the initial 8,951 samples. By the end of the month, the set of samples covered by all 65 lineages grew to 11,877.

Over the course of the month, only 28 samples changed lineages (0.23% of samples), affecting 4 of the 65 designations. auto.XBB.1.5.40 was the primary affected lineage, with 9 samples being added to it and 7 samples being removed, but these 16 samples only constitute 0.7% of the 2119 samples in this large lineage. A full table of samples which change lineages can be found in Supplementary Table 2. Overall, we observe high stability in our lineage designations with regards to samples that remain consistently present on the tree.

However, some of these apparent lineage changes and related stability issues result from duplicated, dropped, or renamed sample data. Several hundred samples were dropped from the global phylogeny on 2023-04-13 and 2023-04-14, affecting 38 of the 65 lineages. In some cases, this resulted in an apparent decline in lineage membership; the lineage designation “auto.XBB.1.29.1” began with 12 samples on 2023-04-01, but declined to only 7 by 2023-04-30. It is likely that the majority of these were replaced with a new name; for example, the sample OX451312.1, a member of auto.XBB.1.29.1, was dropped from the tree on 2023-04-13. Present throughout this period, also within auto.XBB.1.29.1, was the sample Scotland/SCOT-26390/2023|OX451312.1|2023-02-08, with the same tag and date of collection. It’s likely that the inclusion of OX451312.1 represents a spurious duplication of this sample

within the global phylogeny, perhaps because it was uploaded separately to different public databases. Of the 12 original samples of auto.XBB.1.29.1, 5 are dropped on the 13th while matching fuller names with the same collection date and tag information in auto.XBB.1.29.1. Therefore, the original 12 sample set represents an inflated, spurious group and this marginal lineage designation must be dropped or revised. It is worth noting, however, that this lineage remained present throughout the period and the deduplicated samples remained stable members of this group.

SARS-CoV-2 is a densely sampled pathogen and most branches are well supported by many samples worth of data. Additionally, samples incorporated into the SARS-CoV-2 global public phylogenetic trees used for lineage designation are rigorously quality filtered to remove low quality consensus sequences. Generally, lineage designations of more than a few dozen samples are largely stable, with <1% of samples overall changing designations over the course of weeks. Even in the case of recombination, where the base of a lineage cannot be properly represented in a tree, the group itself is generally stable and cohesive. All code for this analysis can be found at https://github.com/jmcbroome/lineage-manuscript.

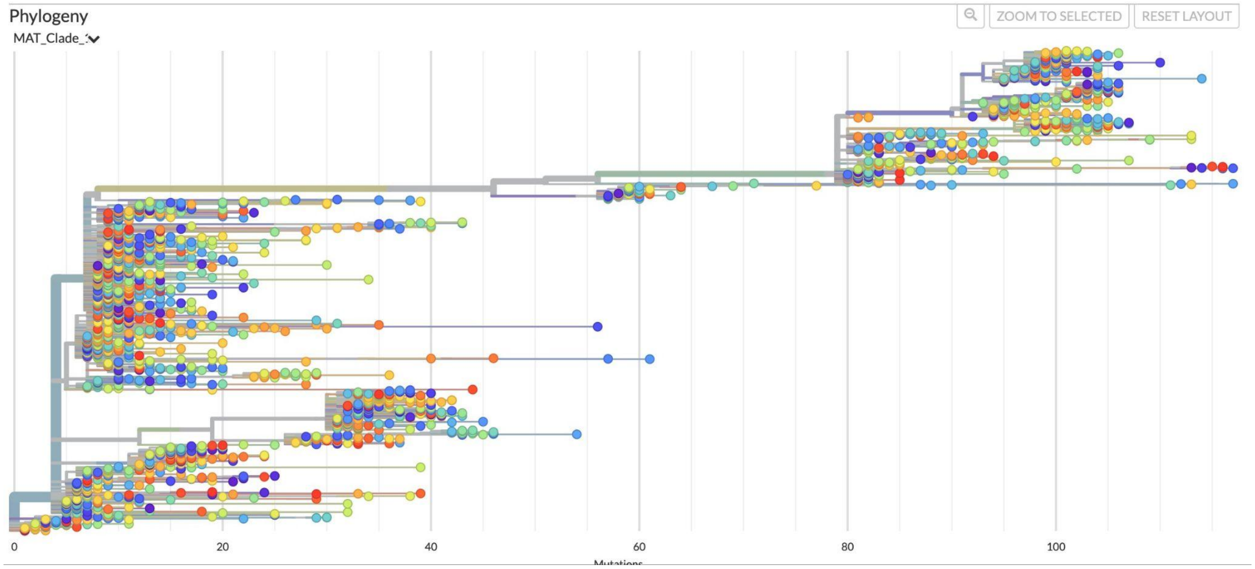

Supplementary Figure 1: Pango Lineage Hierarchy. This figure displays the hierarchical and serial relationships among the defined Pango lineages as of 2022-12-11. Each individually colored tip (dot) on this tree represents a specific Pango lineage.
An interactive version of this figure can be found at https://nextstrain.org/fetch/raw.githubusercontent.com/jmcbroome/lineage-manuscript/main/public-2022-12-11.backbone.json/?c=MAT_Clade_0&tl=MAT_Clade_0

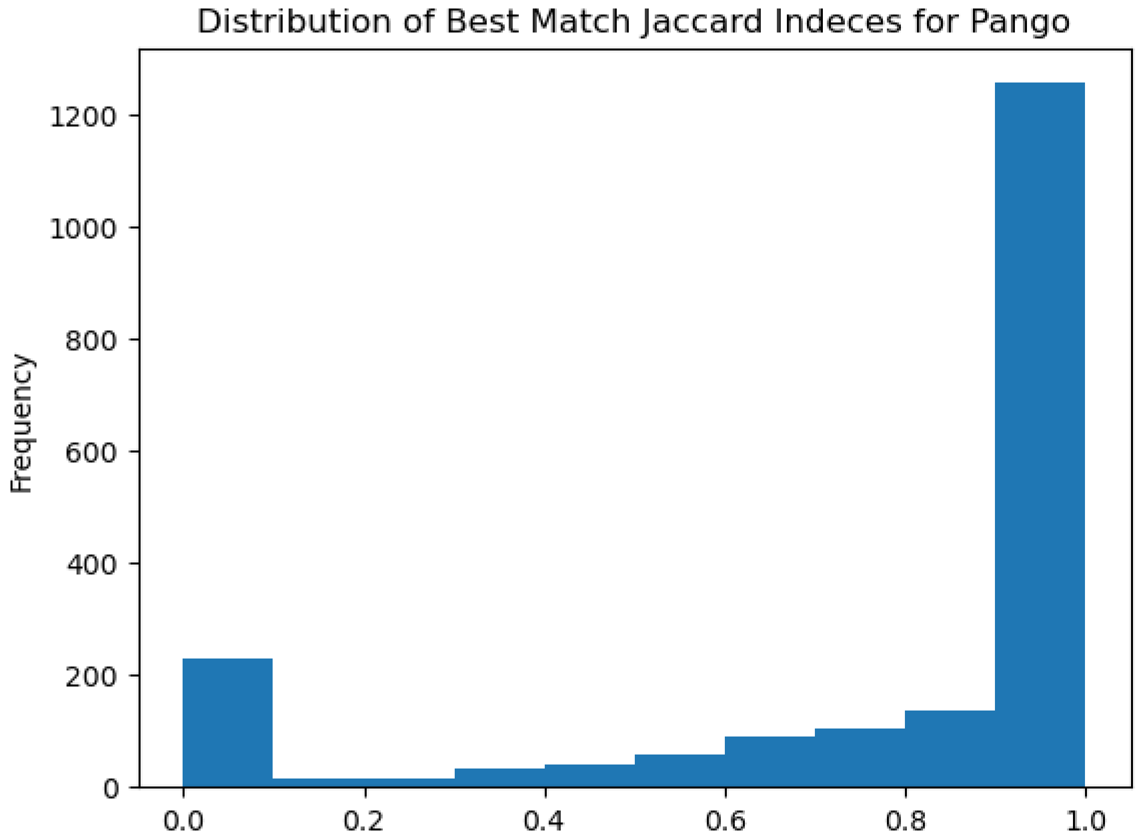

Supplementary Figure 2: Jaccard Index Distribution for Pango Lineages. The distribution is highly bimodal, with a plurality of lineages being perfectly or partially matched by an automatically identified lineage, but with a substantial body of Pango lineages with no strong matching label.

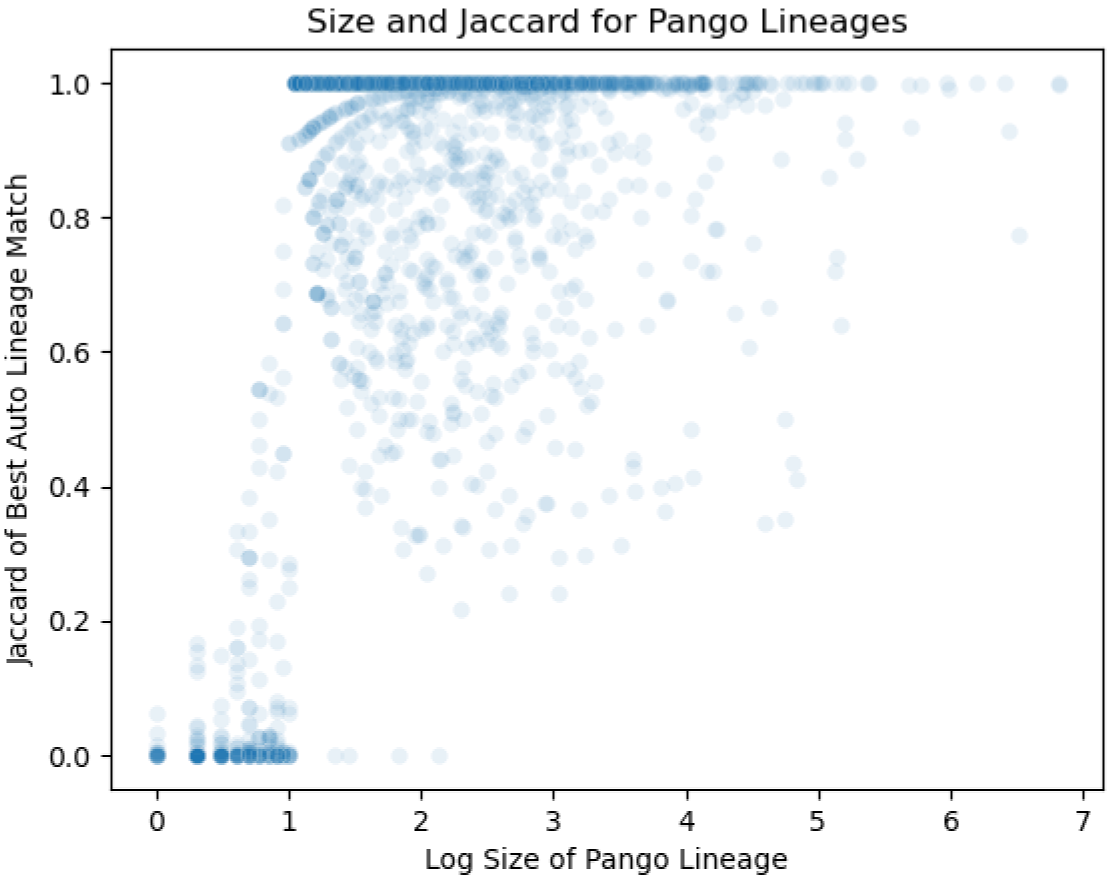

Supplementary Figure 3: Pango Jaccard Indices by Size. We generally find that larger lineages are recaptured well by Autolin. Pango lineages below size 10 (1 on the log10 scale) are poorly recaptured because Autolin filters lineage proposals of less than 10 samples by default.
