# Supplementary material for: A framework for automated scalable designation of viral pathogen lineages from genomic data
Source: Nat Microbiol. 2024 Feb 5;9(2):550–60. doi: 10.1038/s41564-023-01587-5 (PMC10847047; doi:10.1038/s41564-023-01587-5)
Supplement: Supplementary file 2 — Reporting Summary [file 41564_2023_1587_MOESM2_ESM.pdf]

nature portfolio

Corresponding author(s): Jakob McBroome

Last updated by author(s): December 3rd, 2023

Reporting Summary

Nature Portfolio wishes to improve the reproducibility of the work that we publish. This form provides structure for consistency and transparency in reporting. For further information on Nature Portfolio policies, see our Editorial Policies and the Editorial Policy Checklist.

Statistics

For all statistical analyses, confirm that the following items are present in the figure legend, table legend, main text, or Methods section.

n/a	Confirmed	
☐	☒	The exact sample size (*n*) for each experimental group/condition, given as a discrete number and unit of measurement
☐	☒	A statement on whether measurements were taken from distinct samples or whether the same sample was measured repeatedly
☐	☒	The statistical test(s) used AND whether they are one- or two-sided<br>*Only common tests should be described solely by name; describe more complex techniques in the Methods section.*
☒	☐	A description of all covariates tested
☒	☐	A description of any assumptions or corrections, such as tests of normality and adjustment for multiple comparisons
☐	☒	A full description of the statistical parameters including central tendency (e.g. means) or other basic estimates (e.g. regression coefficient) AND variation (e.g. standard deviation) or associated estimates of uncertainty (e.g. confidence intervals)
☐	☒	For null hypothesis testing, the test statistic (e.g. *F*, *t*, *r*) with confidence intervals, effect sizes, degrees of freedom and *P* value noted<br>*Give P values as exact values whenever suitable.*
☐	☒	For Bayesian analysis, information on the choice of priors and Markov chain Monte Carlo settings
☒	☐	For hierarchical and complex designs, identification of the appropriate level for tests and full reporting of outcomes
☒	☐	Estimates of effect sizes (e.g. Cohen's *d*, Pearson's *r*), indicating how they were calculated

Our web collection on statistics for biologists contains articles on many of the points above.

Software and code

Policy information about availability of computer code

Data collection	Our data were provisioned by the UCSC Genomics Institute http://hgdownload.soe.ucsc.edu/goldenPath/wuhCor1/UShER_SARS-CoV-2/ . These datasets were constructed with UShER, filtered with matUtils, and optimized with matOptimize (https://github.com/yatisht/usher). Nextstrain datasets were constructed by the Nextstrain Augur pipeline (using nextstrain-augur v19.1.0, treetime v0.9.4, and iqtree v2.2.0). Additionally, we obtained publicly available data from https://github.com/jbloomlab/SARS-CoV-2-RBD_DMS and downloaded sequence data from GenBank via the Entrez API.
Data analysis	Our primary lineage pipeline for SARS-CoV-2 Pango lineages can be found at https://github.com/jmcbroome/autolin<br>Specific analysis code for the figures and results in this manuscript can be found at https://github.com/jmcbroome/lineage-manuscript<br>The streamlit and CLI tool used for non-sars-cov-2 pathogens can be found at https://github.com/jmcbroome/automated-lineage-json. Other software tools used for visualization include FigTree (v1.4.4), Taxonium, and Nextstrain Auspice. Minimap2 v2.26 was used to align genomic sequence data.

For manuscripts utilizing custom algorithms or software that are central to the research but not yet described in published literature, software must be made available to editors and reviewers. We strongly encourage code deposition in a community repository (e.g. GitHub). See the Nature Portfolio guidelines for submitting code & software for further information.

Data

Policy information about availability of data

All manuscripts must include a data availability statement. This statement should provide the following information, where applicable:

- Accession codes, unique identifiers, or web links for publicly available datasets
- A description of any restrictions on data availability
- For clinical datasets or third party data, please ensure that the statement adheres to our policy

All of our data is publicly available and can be downloaded from http://hgdownload.soe.ucsc.edu/goldenPath/wuhCor1/UShER_SARS-CoV-2/, specifically http://hgdownload.soe.ucsc.edu/goldenPath/wuhCor1/UShER_SARS-CoV-2/2022/12/11/public-2022-12-11.all.masked.pb.gz.
Additional information can be found at https://github.com/jmcbroome/lineage-manuscript.

Human research participants

Policy information about studies involving human research participants and Sex and Gender in Research.

Reporting on sex and gender	n/a
Population characteristics	*Describe the covariate-relevant population characteristics of the human research participants (e.g. age, genotypic information, past and current diagnosis and treatment categories). If you filled out the behavioural & social sciences study design questions and have nothing to add here, write "See above."*
Recruitment	*Describe how participants were recruited. Outline any potential self-selection bias or other biases that may be present and how these are likely to impact results.*
Ethics oversight	*Identify the organization(s) that approved the study protocol.*

Note that full information on the approval of the study protocol must also be provided in the manuscript.

Field-specific reporting

Please select the one below that is the best fit for your research. If you are not sure, read the appropriate sections before making your selection.

☐ Life sciences ☐ Behavioural & social sciences ☒ Ecological, evolutionary & environmental sciences

For a reference copy of the document with all sections, see nature.com/documents/nr-reporting-summary-flat.pdf

Ecological, evolutionary & environmental sciences study design

All studies must disclose on these points even when the disclosure is negative.

Study description	We present a method and example application for the identification of novel SARS-CoV-2 lineages from a global phylogeny.
Research sample	Our dataset is the SARS-CoV-2 global phylogeny of public sequences as of 2023-12-11. This is an appropriate representation of global SARS-CoV-2 genome sequence diversity and serves as the basis for the inference of new SARS-CoV-2 lineages.
Sampling strategy	Our dataset included more than 6 and a half million SARS-CoV-2 genomes from across the entire SARS-CoV-2 pandemic. This is more than sufficient to identify many novel lineages.
Data collection	Our global phylogeny is constructed from whole genome consensus sequences submitted to GISAID and other public repositories by public health groups around the world. These groups generally use the ARTIC primer schema to perform short-read sequencing. These short reads are aligned and a consensus genome is called with a number of different possible tools. Once submitted, the data is publicly available, and we incorporate it into our dataset via UShER.
Timing and spatial scale	SARS-CoV-2 genome sequencing has been occurring since early 2020, though we began constructing our daily global phylogenies in May 2021. Each day, all of the data submitted to Genbank, GISAID and other sources were incorporated into a new daily build. Sequencing and updating of our global phylogenies is ongoing in early 2023, past the date used for the analysis presented in this manuscript.
Data exclusions	We filtered samples from the global tree that are descended from single branches with two or more reversion mutations as being potential consensus construction artifacts, in line with standard practice.
Reproducibility	Our pipeline is deterministic outside of the model fitting step used for sorting, and so a researcher who downloads the same dataset from the public repository and carries out the same analysis we describe in our methods and provision in our associated github repositories will get the same results.

Randomization	The only grouping step is the core lineage inference step. In the case where we statistically compare our generated lineages to a preexisting lineage system, we create a null distribution where equivalent lineages sets are generated at random and evaluate whether our method is significantly more concordant with existing lineages than a random distribution.
Blinding	Blinding does not make sense in this context, as we are using publicly available data and not applying conditions.

Did the study involve field work? ☐ Yes ☒ No

Reporting for specific materials, systems and methods

We require information from authors about some types of materials, experimental systems and methods used in many studies. Here, indicate whether each material, system or method listed is relevant to your study. If you are not sure if a list item applies to your research, read the appropriate section before selecting a response.

Materials & experimental systems

n/a	Involved in the study	
☒	☐	Antibodies
☒	☐	Eukaryotic cell lines
☒	☐	Palaeontology and archaeology
☒	☐	Animals and other organisms
☒	☐	Clinical data
☒	☐	Dual use research of concern

Methods

n/a	Involved in the study	
☒	☐	ChIP-seq
☒	☐	Flow cytometry
☒	☐	MRI-based neuroimaging
